# Supplementary figures and images for: Uncertainty quantification in cerebral circulation simulations focusing on the collateral flow: Surrogate model approach with machine learning
Source: PLoS Comput Biol. 2022 Jul 22;18(7):e1009996. doi: 10.1371/journal.pcbi.1009996 (PMC9307280; doi:10.1371/journal.pcbi.1009996)

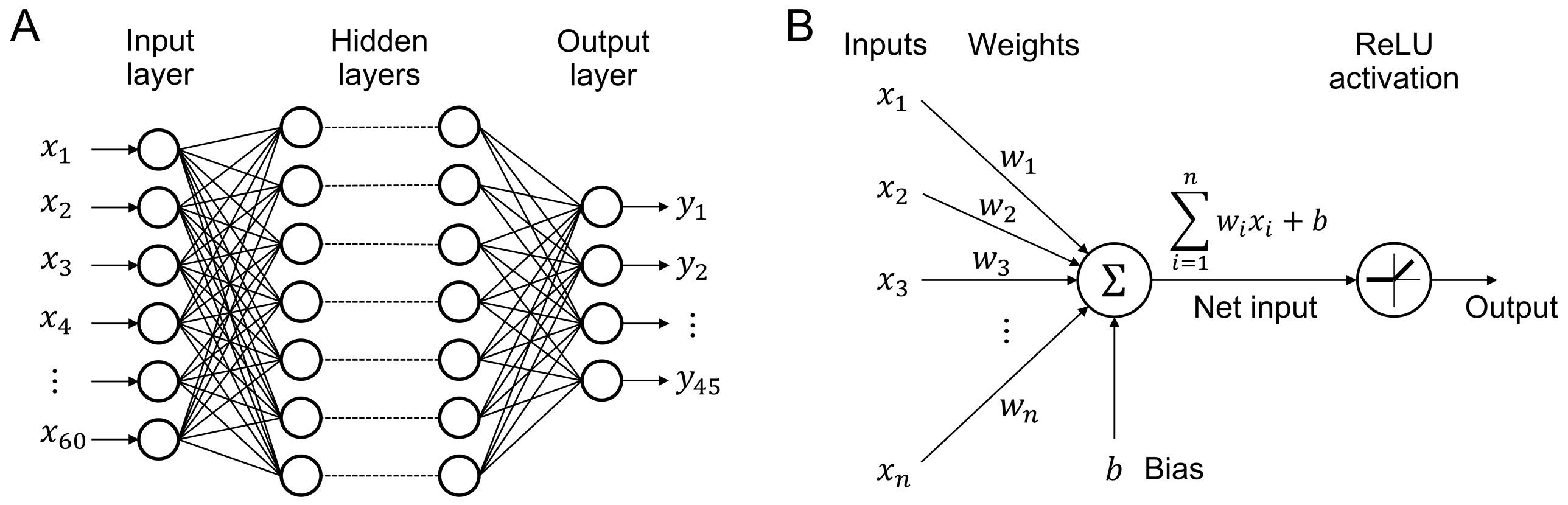

Supplement: S1 Fig — (A) Network architecture. (B) Schematic of a single node. (TIF) [file pcbi.1009996.s003.tif]

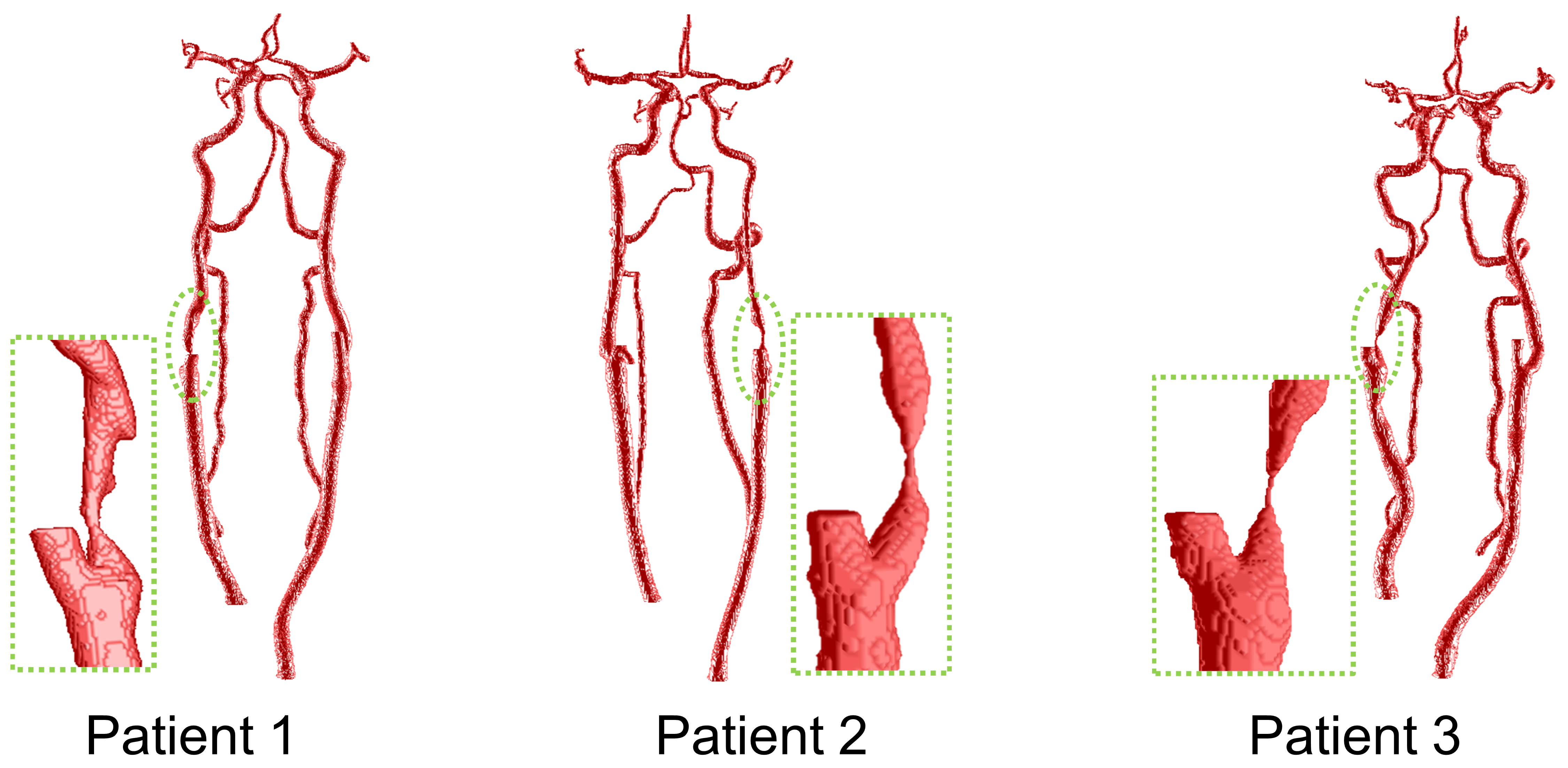

Supplement: S2 Fig — Three-dimensional reconstructed arterial geometries obtained via lumen segmentation on computed tomography images. (TIF) [file pcbi.1009996.s004.tif]

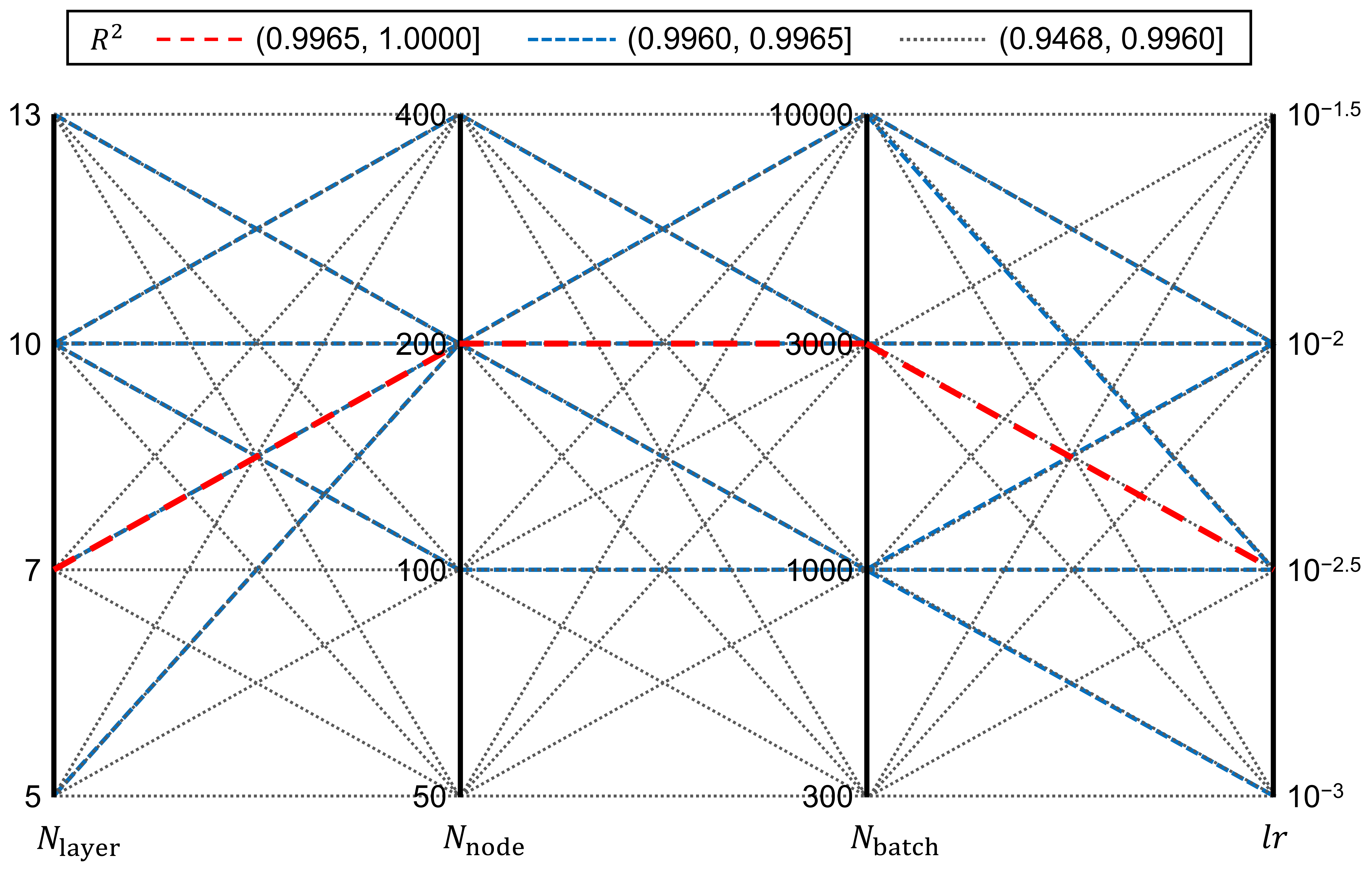

Supplement: S3 Fig — The number of training samples was maintained at 120 000, and the R2 scores were evaluated considering 40 000 test samples. Nlayer denotes the number of hidden layers, Nnode indicates the number of nodes in each hidden layer, Nbatch represents the batch size for mini-batch training, and lr denotes the initial learning rate. (TIF) [file pcbi.1009996.s005.tif]

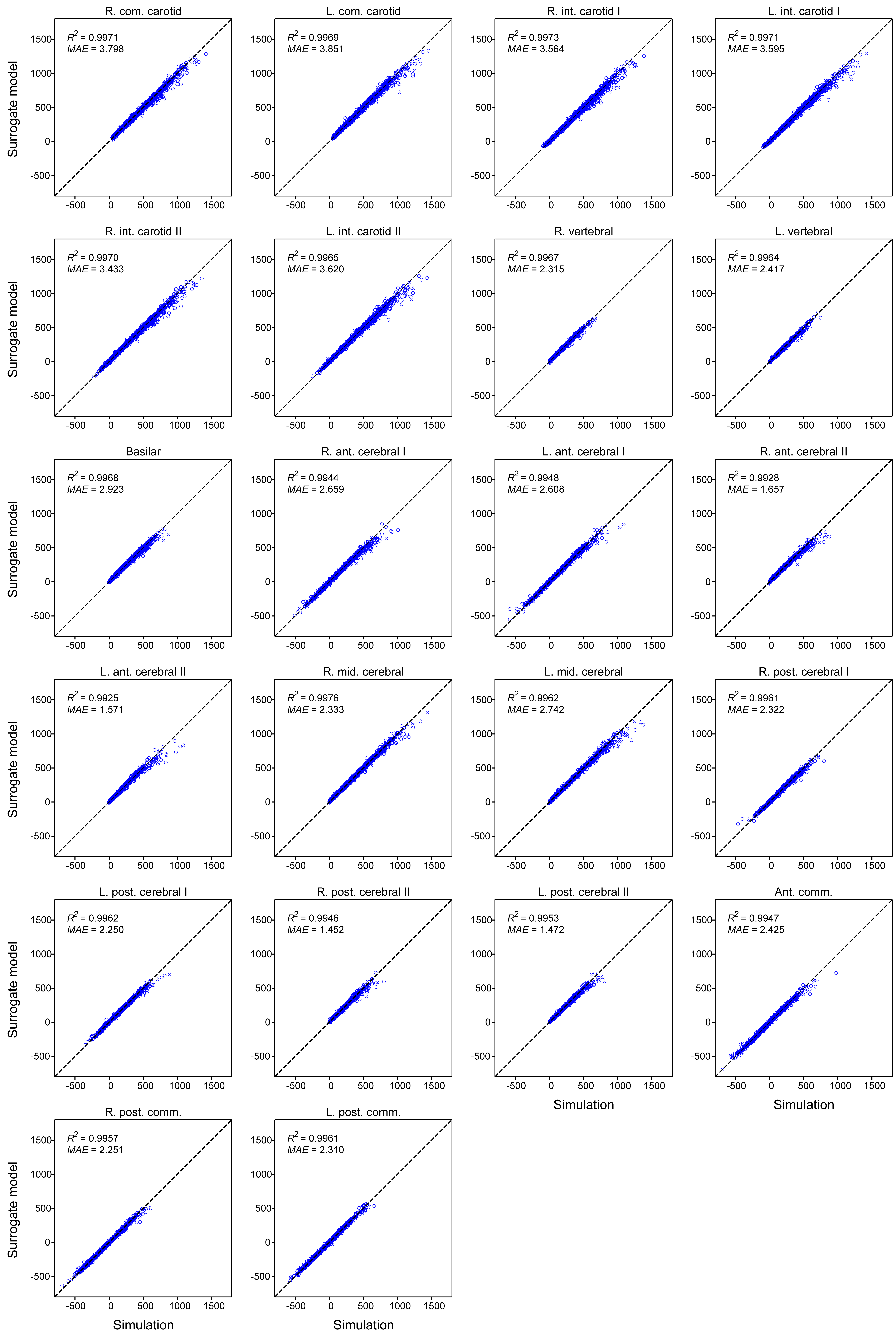

Supplement: S4 Fig — Flow rates in the carotid and cerebral arteries are depicted for 40 000 samples of test data. The negative flow rate indicates that the flow direction is opposite to that of the arrows in Fig 2. The R2 score and mean absolute error (MAE) of each quantity are depicted in the corresponding panels. (TIF) [file pcbi.1009996.s006.tif]

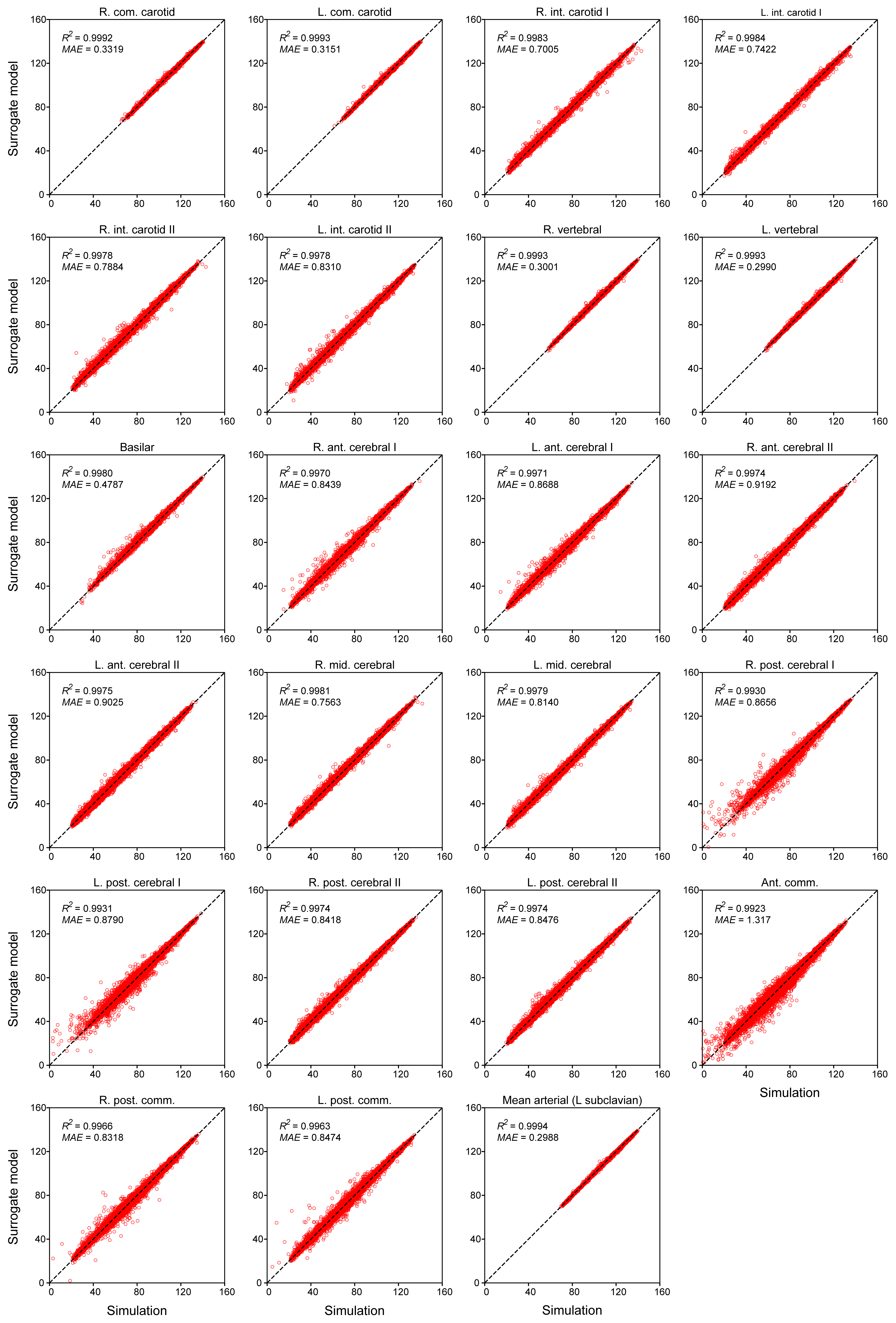

Supplement: S5 Fig — Pressures in the carotid and cerebral arteries are depicted for 40 000 samples of test data. The R2 score and mean absolute error (MAE) of each quantity are depicted in the corresponding panels. (TIF) [file pcbi.1009996.s007.tif]
