# Supplementary material for: Uncertainty quantification in cerebral circulation simulations focusing on the collateral flow: Surrogate model approach with machine learning
Source: PLoS Comput Biol. 2022 Jul 22;18(7):e1009996. doi: 10.1371/journal.pcbi.1009996 (PMC9307280; doi:10.1371/journal.pcbi.1009996)
Supplement: Attachment — Submitted filename: Author_Response_Rev1.pdf [file pcbi.1009996.s008.pdf]

Dear Dr. Beard and Dr. Marsden,

We are pleased to submit the revised version of our manuscript titled "**Uncertainty quantification in cerebral circulation simulations focusing on the collateral flow: Surrogate model approach with machine learning**" to *PLOS Computational Biology*. Please note that we have made minor revisions to the title of our manuscript for conciseness.

We appreciate the time and effort the editors and reviewers have dedicated to providing their insightful feedback on ways to improve our manuscript. All comments have been carefully considered, and the manuscript has been revised accordingly. Please find below the revision note where we have provided point-by-point responses to the reviewer comments. We have attached two versions of the revised manuscript: one with tracked changes denoting where the text has been changed (highlighted version) and a clean version.

We hope that our edits and responses satisfactorily address all reviewer concerns and that the revised manuscript is now suitable for publication in the journal.

We look forward to hearing from you.

Sincerely,
Changyoung Yuhn, PhD

Responses to reviewer comments

We appreciate the reviewers' valuable comments that have helped us improve the manuscript. Please find below our point-by-point responses to the comments (shown in *italics*). The corrections made in the revised manuscript are presented in red font. We have attached two versions of the revised manuscript: one with tracked changes denoting where the text has been changed (highlighted version) and the other a clean version. Note that page numbers, line numbers, and references in this document refer to the **clean version** of the revised manuscript.

Please note that the title of our manuscript has been changed to "Uncertainty quantification in ~~the~~ cerebral circulation simulations focusing on the collateral flow: Surrogate model approach with machine learning" to remove unnecessary 'the' and shorten the title.

Response to Reviewer #1

The authors propose a ML algorithm based on neural networks to efficiently estimate the patient-specific risk of cerebral hyperperfusion (CH) using uncertainty quantification (UQ). Clinical intervention is sometimes required in the presence of stenoses affecting the right or left carotid arteries. Cerebral hyperperfusion occurs when an increase of more than 100% in the circle of Willis flow rate is observed post intervention. The authors train a neural network to map 60 parameters describing the geometry of cerebral arteries and stenoses to 45 time-

averaged quantities (flow rate and pressures). The gain in runtime with respect to 0D-1D reduced order models is dramatic (milliseconds vs minutes) and the possibility of parallelization makes this method attractive for UQ in clinical settings.

Overall, the methods discussed in this paper are scientifically sound and the strenght/limitations of the approach are clearly presented. Below are some minor remarks and observations that I believe should be addressed prior to publication.

Response: We appreciate the time and effort you have dedicated to providing your thoughtful comments and suggestions.

1) Fig. 1 could be improved by including a brief description of the inputs (¥mathbf{x}) and the outputs (¥mathbf{y}_{sim}) either in the figure itself or in the caption; "sampling inputs that represent the anatomical and physiological conditions and collecting the corresponding simulation outputs" is too general.

Response: We agree with your suggestion and have therefore added a brief description of the inputs ($\boldsymbol{x}$) and the outputs ($\boldsymbol{y}_{\mathrm{sim}}$) to the figure caption.

- Page 7, lines 163–166 (Fig 1 caption): "The datasets were generated by randomly sampling 60 inputs (column vector $\boldsymbol{x} \in \mathbb{R}^{60}$) describing the geometry of cerebral arteries and stenoses, and collecting the corresponding 45 simulation outputs (column vector $\boldsymbol{y}_{\mathrm{sim}} \in \mathbb{R}^{45}$) of time-averaged flow rates and pressures."

2) Page 12, line 253: the authors mention that they use the Newton-Raphson method to enforce conservation of mass and total pressure at junctions. However, we use the Newton-Raphson to solve nonlinear equations, and these constraints are linear. I believe that the use of the Newton-Raphson method is necessary due to the presence of stenoses models which are nonlinear. I recommend clarifying this point.

Response: Thank you for this insightful comment. As you pointed out, the Newton–Raphson method was used to solve nonlinear equations. Let us explain here that not only the coupling of the stenosis model but the constraints of the conservation of mass and total pressure at bifurcations are nonlinear as well.

Let us consider bifurcating arteries as shown in Fig R1. The conservation of mass and total pressure at bifurcations are given as follows [41]:

$$Q_{\mathrm{m}}^{n} = Q_{\mathrm{d1}}^{n} + Q_{\mathrm{d2}}^{n}, \tag{R1}$$

$$P_{\mathrm{m}}^{n} + \frac{\rho}{2}\left(\frac{Q_{\mathrm{m}}^{n}}{A_{\mathrm{m}}^{n}}\right)^{2} = P_{\mathrm{d1}}^{n} + \frac{\rho}{2}\left(\frac{Q_{\mathrm{d1}}^{n}}{A_{\mathrm{d1}}^{n}}\right)^{2}, \tag{R2}$$

$$P_{\mathrm{m}}^{n} + \frac{\rho}{2}\left(\frac{Q_{\mathrm{m}}^{n}}{A_{\mathrm{m}}^{n}}\right)^{2} = P_{\mathrm{d2}}^{n} + \frac{\rho}{2}\left(\frac{Q_{\mathrm{d2}}^{n}}{A_{\mathrm{d2}}^{n}}\right)^{2}, \tag{R3}$$

where Q^n, A^n, and P^n denote the volumetric flow rate, cross-sectional area of the artery, and internal pressure at the n-th time step (i.e., at $t = n\Delta t$), respectively; the subscript 'm' denotes the quantities at the last grid point of mother artery; and 'd1' and 'd2' the quantities at the first

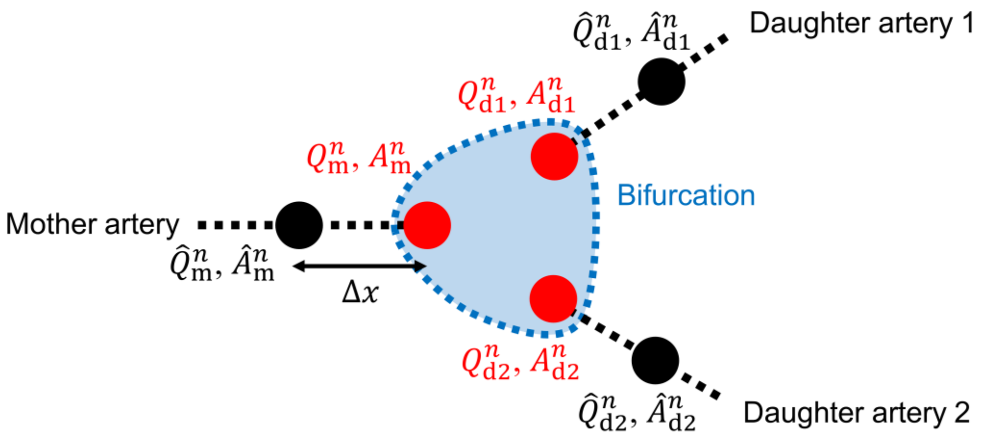

Fig R1. One-dimensional arteries at the bifurcation.

grid point of two daughter arteries. In Equations (R1)–(R3), we have six unknowns: Q^n_m, Q^n_{d1}, Q^n_{d2}, A^n_m, A^n_{d1}, and A^n_{d2}. To close the system, we obtain three more equations from the conservation of mass between two grid points close to the bifurcation in each artery [41]:

$$\frac{\frac{\hat{A}^n_m + A^n_m}{2} - \frac{\hat{A}^{n-1}_m + A^{n-1}_m}{2}}{\Delta t} + \frac{Q^n_m - \hat{Q}^n_m}{\Delta x} = 0, \tag{R4}$$

$$\frac{\frac{A^n_{d1} + \hat{A}^n_{d1}}{2} - \frac{A^{n-1}_{d1} + \hat{A}^{n-1}_{d1}}{2}}{\Delta t} + \frac{\hat{Q}^n_{d1} - Q^n_{d1}}{\Delta x} = 0, \tag{R5}$$

$$\frac{\frac{A^n_{d2} + \hat{A}^n_{d2}}{2} - \frac{A^{n-1}_{d2} + \hat{A}^{n-1}_{d2}}{2}}{\Delta t} + \frac{\hat{Q}^n_{d2} - Q^n_{d2}}{\Delta x} = 0, \tag{R6}$$

where $\hat{Q}^n$ and $\hat{A}^n$ denote the volumetric flow rate and cross-sectional area of the artery at the neighboring grid point of the bifurcation point (Fig R1). Substituting Equations (R4)–(R6) into Equations (R1)–(R3) yields the coupled nonlinear equations. To solve them, we used the Newton–Raphson method.

We acknowledge that the original sentence was unclear regarding the use of the Newton–Raphson method. For clarification, we have now explained in the manuscript that enforcing the conservation of mass and total pressure at bifurcations yields the coupled nonlinear equations.

- Page 11, lines 259–262: "Bifurcated 1D segments were coupled by enforcing the conservation of mass and total pressure at the bifurcations. As this yields the coupled nonlinear equations (see [41] for detailed formulas), we used the iterative Newton–Raphson method to solve them [6, 41]."

3) Page 12, line 294: "within a reasonable range": the authors could add here that this aspect will be discussed later on in the paper (in paragraph Design of experiments). The reader might be confused by the use of "reasonable" here without further explanation

Response: We agree with your suggestion and have explained in the revised manuscript that the detailed aspect will be discussed later in the "Design of experiments" section.

- Page 12, lines 301–303: "(…) and were reproduced by randomly sampling 60 input parameters within a reasonable range (as will be discussed later in the "Design of experiments" subsection)."

4) Page 13, line 301: there's an unmatched "(" in this sentence.

Response: We apologize for this typo. We have removed the unmatched left bracket before "in."

- Page 12, line 310: "Diameters of 22 carotid and cerebral arteries in the 1D model (22 parameters)"

5) Page 13, lines 311-314: "The variation...as indicated in Equation (5)". These sentences are a bit unclear to me. Are the authors saying that Ls should also be varied because Rv depends on it, but they take it constant because the third term in Eq.(4) is negligible? If so, please rephrase to make this point clearer.

Response: We apologize for this confusion, and the text has been revised accordingly. We hope this clarifies why we did not select the stenosis length L_s as input.

- Page 13, lines 320–325: "Note that we do not select the stenosis length, L_s, as an input. As shown in Equation (4), L_s has two effects on ΔP: one on the third term and the other on the first term via R_v. The effect of L_s on the third term can be ignored because the third term is negligible compared to the other terms. Furthermore, since we selected R_v as the input representative of the stenosis geometry, encompassing the variations of $D(x)$ and L_s, it is unnecessary to select L_s as a separate input to be varied."

6) Page 17, Eq.(9) does the last layer feature a ReLu activation function? Isn't this incompatible with the type of normalization used for the outputs (standard normalization, discussed at page 19), meaning that negative values will never be predicted by the neural network?

Response: We apologize for this confusion. The ReLu activation function was used, except for the connection between the hidden and output layers. Thus, the output from the neural network can take a negative value. We have corrected the explanation regarding DNN and Equation (11).

- Page 16 lines 385–386: "The DNN comprises a total of $N_{\text{layer}} + 2$ layers: an input layer, a series of N_{layer} hidden layers, and an output layer (S1 Fig)."
- Page 16, lines 391–393: "This process continues for each layer up to the last hidden layer. The nodes in the last hidden layer and the output layer are fully connected without ReLU activation."
- Page 17, Equation (11):

$$\boldsymbol{y}^l = \begin{cases} \boldsymbol{x}, & (l = 1) \\ \max(0, \boldsymbol{W}^l \boldsymbol{y}^{l-1} + \boldsymbol{b}^l), & (2 \le l \le N_{\text{layer}} + 1) \\ \boldsymbol{W}^l \boldsymbol{y}^{l-1} + \boldsymbol{b}^l, & (l = N_{\text{layer}} + 2) \end{cases} \quad (11)$$

7) Page 20, line 430: is Rv computed using Eq.(5)? If so, please refer to it for clarity.

Response: Yes, the values of R_v were computed using Equation (5). We have added reference to Equation (5) in the text.

- Page 19, line 455: "(…) and the corresponding R_v values (evaluated using Equation (5)) were 0.5 mmHg s mL^{-1}, (…)."

8) Page 22, line 492: Please expand the title of the paragraph: SA -> Stability Analysis.

Response: We agree that an acronym should be avoided in section headings unless it is very well-known. We have expanded all such acronyms across the paper.

- Page 16, line 384: "DNN" → "Deep neural network"
- Page 18, line 440: "UQ and SA" → "Uncertainty quantification and sensitivity analysis"
- Page 21, line 524: "SA" → "Sensitivity analysis"
- Page 24, line 610: "UQ and SA" → "Uncertainty quantification and sensitivity analysis"
- Page 28, line 722: "Surrogate modeling approach for the UQ" → "Surrogate modeling approach for uncertainty quantification"
- Page 31, line 800: "Biological implications: CH and collateral circulation" → "Biological implications: Cerebral hyperperfusion and collateral circulation"

9) Page 23, Eq.(16): I am not sure that the upper bound in the sum is correct. If Nlayer = 1, the sum contains two terms as if the number of layers is actually two. Perhaps this is a problem of notation and Nlayer only refers to the hidden layers, whereas the authors consider the output layer a separate one.

Response: We apologize for this confusion. As you mentioned, N_layer refers to the number of hidden layers; the DNN thus has $N_\mathrm{layer} + 2$ layers in total. Since the input layer has no parameters (it just passes inputs to the first hidden layer), the number of layers that contains parameters is $N_\mathrm{layer} + 1$. Therefore, the number of indices for the summation in Equation (18) should be $N_\mathrm{layer} + 1$, which is correct as it is. Nevertheless, we have revised the starting and ending indexes of the summation to avoid confusion. Please also see the revised Equation (11).

- Page 22, Equation (18):

$$N_\mathrm{param} = \sum_{l=2}^{N_\mathrm{layer}+2} \left(N_\mathrm{node}^{l} \cdot N_\mathrm{node}^{l-1} + N_\mathrm{node}^{l}\right), \tag{18}$$

- Page 22, lines 548–549: "Note that the index of summation starts at 2 instead of 1 since the input layer (l = 1) has no parameters."

10) Page 26: when discussing the parallelization, the authors could say a few words on how this was implemented. Are they launching one "simulation" at the time but using the GPU to

optimize the matrix-matrix multiplications? Or are they launching multiple threads each performing a single simulation by exploiting the fact that the simulations are independent? In the latter case, it might be worth noting that the same could be done for the 1D-0D, provided that each process/thread have access to sufficient memory.

Response: Thank you for the suggestion. The parallelization we discuss on page 24, lines 598–602 refers to the former method and not the latter ("data-parallel method"). For parallelization, we used the method of launching a single process and performing CUDA-based parallel matrix operations on a GPU. We used the built-in backend of Chainer [61] to implement parallelization on a GPU. The latest deep learning libraries, including TensorFlow, Keras, PyTorch, and Chainer, support GPU backends and have extensive documentation. As suggested, we have added a brief explanation regarding GPU execution (parallelization).

- Page 24, lines 602–606: "Parallelization on the GPU was performed using the built-in backend of Chainer [61] for CUDA-based parallel matrix operations. The latest deep learning libraries, including TensorFlow, Keras, PyTorch, and Chainer, support GPU execution using their built-in backends, allowing easy parallelization of matrix operations in training and predictions."

11) Fig. 8: what is the meaning of negative ¥Delta ¥overbar Q in each of the distributions? Should negative values be considered not physiological? Please clarify in text when discussing this plot.

Response: Thank you for bringing this up. A negative $\Delta\bar{Q}$ (observed in 1.3%, 0.9%, and 0.1% of the Monte Carlo samples of Patients 1, 2, and 3, respectively) indicates that the flow rate in the middle cerebral artery on the stenosis side decreased immediately after the dilation of stenosis compared to the preoperative value, as per definition (page 19, Equation (15)). This situation may be rare in actual patients with severe stenosis; nonetheless, it is not non-physiological, as observed in some clinical cases (Fig 1 in [36]).

In accordance with your comment, we have clarified the meaning of negative $\Delta\bar{Q}$ in the revised manuscript.

- Page 25, lines 623–625: "A negative $\Delta\bar{Q}$ indicates a decrease in the flow rate following the surgery. This situation may be rare in actual patients with severe stenosis; nonetheless, it is not non-physiological, as observed in certain clinical cases [36]."
- Fig 9: Axis values "0" and ">100" of the color bar have been corrected to "≤0" and "≥100," respectively.

12) Page 28, line 598: "The distribution of ¥Delta ¥overbar Q..." are the authors suggesting that more severe stenoses are associated with more uncertainty? Can they give an explanation of why this is the case?

Response: You have raised an important question. As you pointed out, more severe stenoses were associated with more uncertainty (larger variation) in $\Delta\bar{Q}$ (Fig 8). This trend is attributed to the 2-pixel uncertainty (page 19, lines 470–476) considered for the stenosis geometry. As indicated by Equation (5), the viscous resistance of the stenosis (R_{v}) is inversely proportional to the fourth power of the diameter. This means that the uncertainty of R_{v} increases with a

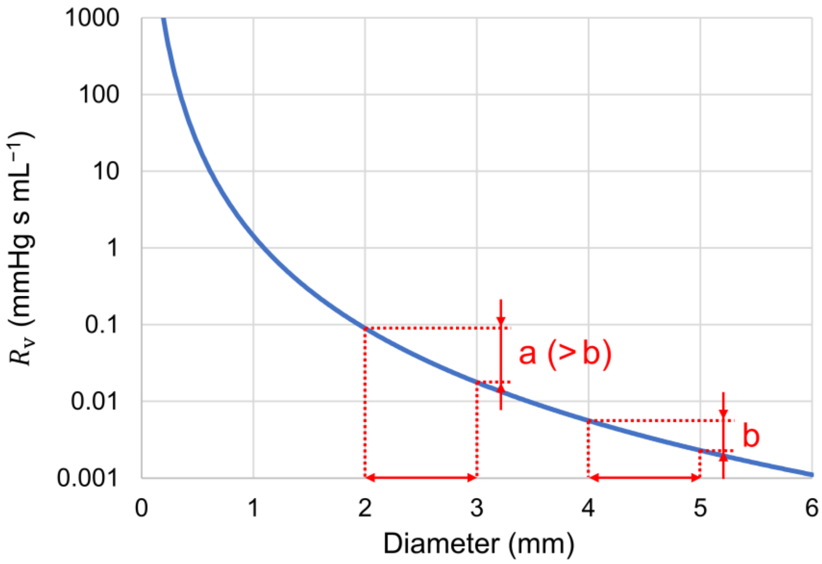

Fig R2. Relationship between viscous resistance (R_v) and arterial diameter. The relationship given by Equation (5) is depicted assuming geometry with a constant diameter and a unit length.

smaller diameter, given the same variation width of diameter (Fig R2). Similarly, the stenosis ratio (SR) exhibits a wider range of variation as the stenosis is more severe. The large uncertainty in R_v and SR would have resulted in large uncertainty in the $\Delta\bar{Q}$ prediction.

However, a comparison of Patients 2 and 3 shows that whether $\Delta\bar{Q}$ exceeds 100% is a separate issue from the amount of uncertainty in $\Delta\bar{Q}$. This is the reason why we proceeded to investigate (i) the conditions that resulted in $\Delta\bar{Q} > 100\%$ and (ii) parameter sensitivities to $\Delta\bar{Q}$ in the subsections "Patient conditions causing cerebral hyperperfusion" and "Sensitivity of uncertain parameters."

We have revised the text to reflect the above information and to improve the information flow.

- Page 25, lines 635–645: "The increase in the prediction uncertainty in $\Delta\bar{Q}$ with higher stenosis severity is attributed to the 2-pixel uncertainty considered for the arterial diameter. With the same variation width of diameter, the uncertainty in R_v (Equation (5)) and SR ($= 1 - D_\mathrm{s}/D_\mathrm{n}$) increases with a smaller diameter, leading to a larger uncertainty in $\Delta\bar{Q}$. However, the comparison of Patients 2 and 3 indicated that CH ($\Delta\bar{Q} > 100\%$) is not caused solely by the severity of stenosis. Patient 2 exhibited a 3.8% chance of CH, whereas the corresponding estimates for Patients 1 and 3 were 0% and 0.001% (only one sample out of 100 000 samples), respectively. In Patient 2, who was assumed to have a possible missing ACoA, the variability of $\Delta\bar{Q}$ to values above 100% was prominent compared to Patient 3, implying that $\Delta\bar{Q}$ was significantly affected by this artery."

13) Page 31, line 633: Can the authors explain more clearly what they mean by vertical and horizontal variations?

Response: We have rewritten the paragraph and hope that the revised text clarifies the meaning of vertical and horizontal variations of flow rates in Fig 10.

- Page 26, lines 671–682: "Fig 10 depicts the variation in the preoperative flow rate in the ACoA (left column) and PCoA (right column) with respect to the diameter. Note that the flow rate shown in Fig 10 varies both horizontally and vertically. As indicated by the relationship between ΔP and $R_v Q$ in Equation (4), the flow rate in an artery is proportional to the pressure difference between the two ends and is inversely proportional to the fourth power of the diameter. The horizontal variation in flow rate shown in Fig 10 is attributed to the diameter variation of the communicating artery within the uncertainty range. On the contrary, the vertical variation is caused by variations in the pressure difference between the ends (i.e., the pressure difference between arteries on the normal and stenosis sides) resulting from uncertainties in the diameter of other arteries, stenosis severity, and flow measurements. As seen from the large vertical variations, the flow rate of the communicating artery is strongly influenced not only by the diameter uncertainty of this artery but also by other uncertainties."

We wish to thank you again for your valuable comments on our manuscript. We hope that we have satisfactorily addressed all your issues and concerns.

Response to Reviewer #2

General comments:

This paper presents a novel systematic framework to evaluate collateral circulation in the circle of Willis (CoW) using a machine-learning-based surrogate model for blood circulation. The hemodynamic data in the surrogate model show reasonable correspondences with those in an original 0D-1D hemodynamic model, and the computational cost of the surrogate model is much less than that of the original hemodynamic model. This enables to reasonably perform uncertainty quantification (UQ) and sensitivity analysis (SA) focusing on some uncertainly geometrical and functional parameters in the analyses. Three patient-specific data are used for the UQ and SA, and risks of cerebral hyperperfusion are discussed with features of collateral flows in the CoW.

Since the approach is excellent and the obtained results and discussion are reasonable, I think the paper is worth being published in this journal. I nonetheless have a few unclear points listed below, so I would appreciate it if the authors clarify and discuss them.

Response: We appreciate the time and effort you have dedicated to providing your constructive comments and suggestions.

Specific comments:

Page 14 – The authors state that the variation of stenosis length Ls is ignored, but also state that the effect of Ls is reflected in Rv in equation (5). This confuses me because the changing Rv is attributed to the change of Ls. Why did the authors directly vary Rv instead of Ls?

Response: We apologize for this confusion, and the text has been revised accordingly. We hope this clarifies why we did not select the stenosis length L_s as input.

- Page 13, lines 320–325: "Note that we do not select the stenosis length, L_s, as an input. As shown in Equation (4), L_s has two effects on ΔP: one on the third term and the other on the first term via R_v. The effect of L_s on the third term can be ignored because the third term is negligible compared to the other terms. Furthermore, since we selected R_v as the input representative of the stenosis geometry, encompassing the variations of $D(x)$ and L_s, it is unnecessary to select L_s as a separate input to be varied."

Page 14 – The surrogate model predicts cycle-averaged hemodynamic quantities, whereas the original 1D model provides a spatial profile in each vessel. Is the cycle-averaged in the surrogate model also indicate the spatial average in each vessel?

Response: Yes, the cycle-averaged flow rate and pressure predicted by the surrogate model can be viewed as axially averaged quantities in each artery. Based on the 1D–0D simulation, we can obtain $A(t,x)$, $Q(t,x)$, and $P(t,x)$ at each axially aligned grid point of the 1D artery. In other words, the 1D model provides axial profiles of A, Q, and P as well as their time variation. Since we are interested in the time-averaged quantities of flow rate and pressure, we take the average of $Q(t,x)$ and $P(t,x)$ with time over the cardiac cycle duration, which we refer to as cycle-averaged flow rate and pressure:

$$\bar{Q}(x) = \frac{1}{T_c}\int_{t_s}^{t_s+T_c} Q(t,x)\,dt\,, \tag{R7}$$

$$\bar{P}(x) = \frac{1}{T_c}\int_{t_s}^{t_s+T_c} P(t,x)\,dt\,, \tag{R8}$$

where t_s and T_c respectively denote the time to start averaging and the cardiac cycle duration (fixed at 1 s). By linearizing Equations (1)–(3) and integrating with x and t (consider integrating Equations (6) and (7) with t over a cardiac duration, we can obtain a simplified relation of $\bar{Q}$ and $\bar{P}$ at adjacent i-th and $(i+1)$-th grid points:

$$\bar{Q}_{i+1} - \bar{Q}_i = 0, \tag{R9}$$

$$\bar{P}_{i+1} - \bar{P}_i = -\frac{22\pi\mu\Delta x}{\bar{A}_{i+1}^2}, \tag{R10}$$

where Δx is the grid spacing. From Equations (R9) and (R10), we see that (i) $\bar{Q}$ is constant in the axial direction and (ii) $\bar{P}$ decreases almost linearly (unless there is a significant change in $\bar{A}$) in the axial direction. As we considered $\bar{Q}$ and $\bar{P}$ at the grid point in the middle of each artery as the outputs, $\bar{Q}$ and $\bar{P}$ predicted by the surrogate model can be viewed as axially averaged quantities in each artery.

For clarity, we have revised the corresponding text in the manuscript as follows:

- Page 13, lines 326–327: "Based on the 1D–0D simulation, $A(t,x)$, $Q(t,x)$, and $P(t,x)$ at each axially aligned grid point of the 1D artery can be obtained as the output."
- Page 13, lines 338–343: "Here, cycle-averaged flow rate and pressure refer to Q and P averaged over a cardiac cycle duration:

$$\bar{Q}(x) = \frac{1}{T_c}\int_{t_s}^{t_s+T_c} Q(t,x)\,dt\,, \tag{9}$$

$$\bar{P}(x) = \frac{1}{T_c}\int_{t_s}^{t_s+T_c} P(t,x)\,dt\,, \tag{10}$$

where t_s and T_c respectively denote the time to start averaging and cardiac cycle duration (fixed as 1 s). In the axial direction, $\bar{Q}$ is constant and $\bar{P}$ decreases almost linearly unless there is a significant axial change in $\bar{A}$. Therefore, $\bar{Q}$ and $\bar{P}$ at the middle grid point of each artery can be regarded as the axially averaged quantities in each artery."

Table 2 – It is not clear to me what the boundary condition is imposed on the 0D-1D and surrogate model and how the value is varied in the UQ (what quantity does in Table 2 reflects the boundary condition?).

Response: Thank you for bringing this up. Peripheral resistances (peripheral resistances at the six outlets of the circle of Willis and scaling factor for the total peripheral resistance) in Table 2 reflect the boundary condition to the 1D arterial network.

In the 1D–0D simulation, we coupled the 1D model with the 0D closed-loop model to set boundary conditions for the starting and terminal grid points of the 1D arterial network. The 0D closed-loop model comprises the peripheral artery, upper and lower body blocks, and heart.

> Page 9, lines 207–209: "The inlet and outlet boundary conditions for the 1D network were obtained by coupling the network with the 0D closed-loop model, which represents the peripheral circulation and heart as lumped parameter networks."

> Page 10, lines 247–252: "The 0D closed-loop model comprises the peripheral artery, upper and lower body blocks, and heart (Fig 2). The peripheral arteries distal to the 1D terminal arteries are represented by the three-element Windkessel model (RCR circuit). In each upper or lower body block, the capillaries, venules, and veins are modeled as RLC circuits in series. The heart is modeled based on the time-varying elastance method, which provides the inlet boundary condition to the 1D network, generating a closed-loop system."

- Page 9, lines 217–219 (Fig 2 caption): "The inlet and outlet boundary conditions for the 1D network are obtained by coupling with the 0D closed-loop model, which represents the peripheral circulation and heart."

Although the 0D model includes a large number of parameters, only some have a significant effect on the cycle-averaged flow rates and pressures in the cerebral arteries, and those are peripheral resistances in Table 2.

- Page 12, lines 306–309: "Although the 1D–0D model includes a large number of parameters, only some have a significant impact on cerebral circulation. As described in the "Patient-specific modeling" subsection, we set those parameters in a patient-specific manner based on the patients' clinical data. The parameters include (…)"

In the 1D–0D simulation, we set peripheral resistances in a patient-specific manner based on the measured flow rate and pressure data, as described in the "Patient-specific modeling"

subsection. The concept of "boundary condition" does not apply to the surrogate model because it only relates inputs to outputs rather than solving governing equations given boundary conditions. The surrogate model directly relates the inputs in Table 2 (including peripheral resistances) to the cycle-averaged flow rates and pressures in the cerebral arteries, as shown in Equation (11).

In the uncertainty quantification, we calibrated the peripheral resistances fed to the surrogate model as we did in the 1D–0D simulation. That is, in the "preoperative adjustment" process (Fig 4), the surrogate model initially predicts the flow rate given literature values of peripheral resistances, the predicted flow rates are compared to the measured flow rates (which are varied considering the uncertainty), and peripheral resistances are updated based on the flow rate differences, and so on until the predicted flow rates and measured flow rates match.

> Page 20, lines 495–499: "In each realization, uncertain inputs and targets were sampled from a specified probability distribution, and $\Delta\bar{Q}_i$ was predicted through successive steps of "preoperative adjustment" and "postoperative prediction" (Fig 4). In the first step, the PRs of the CoW and scaling factor for the total PR were adjusted to match the predicted outputs to the targets. The samples were rejected if target convergence was not attained."

We have added supporting information "S2 Appendix" for a detailed explanation of the uncertainty propagation method.

- Page 21, lines 508–509: "A detailed description of the algorithm for uncertainty propagation is provided in S2 Appendix."
- Page 21, lines 516–517 (Fig 4 caption): "Additional details regarding the algorithm are provided in S2 Appendix."
- Added S2 Appendix "Details on the uncertainty propagation."

We hope that the edited text answers your question clearly.

Page 19 – The authors normalize the training data being [-1,1] to improve the model performance. Is this a standard process for the DNN used in this study? I would appreciate it if the authors more clarify this point.

Response: We appreciate your comment on this point. The min-max normalization applied to the inputs and the standardization (or z-score normalization) applied to the outputs are standard preprocessing in supervised learning. In min-max normalization, both [0, 1] and [−1, 1] are used as typical final intervals. We have added an explanation and a new reference [62] to the text.

- Page 18, lines 429–431: "The normalization and standardization (also known as z-score normalization) applied to the data herein constitute standard preprocessing in supervised learning [62]."
- 62. García S, Luengo J, Herrera F. Data preparation basic models. In: Data preprocessing in data mining. Cham: Springer; 2015. pp. 39–57. https://doi.org/10.1007/978-3-319-10247-4_3

Fig. 4 – I could not understand the meaning of the "statistics converged" process in the "postoperative prediction" and the necessity for going back to the "Monte Carlo sampling" process under un-converged. Why does the post(-operative) process affect the pre(-operative) one?

Response: We apologize for the lack of explanation. The Monte Carlo method requires as many samples as the probability distribution converges (i.e., increasing the number of samples does not change the distribution). Since the appropriate number of samples depends on the case, the number of samples should be determined based on the arbitrary convergence criterion. In Fig 4, the "statistics converged" process indicates a convergence check, and going back to the "Monte Carlo sampling" indicates adding the number of samples. We have revised the text, figure, and figure caption as follows.

- Page 20, lines 505–508: "N_{MC} was increased sequentially until the statistics of $\Delta\bar{Q}_i$ converged. As a basic policy, we increased N_{MC} by 10 000 and ensured that the change in mean and variance of $\Delta\bar{Q}_i$ was within 0.1%. We also confirmed that there was no significant change in the probability of $\Delta\bar{Q}_i > 100\%$ when N_{MC} was increased."
- Page 21, line 514 (Fig 4 caption): "The number of samples was increased sequentially until the statistics converged."
- Fig 4: Added "Add samples" to the figure.

We wish to thank you again for your comments and trust that the revised manuscript is now suitable for publication.

Other minor changes

Although not requested by the reviewers, we have made the following corrections.

- Page 8, lines 188–193: The full names of the Ethics Committees have been provided in the ethics statement.

  Original "Patient data were collected and provided by the Rakuwakai Otowa Hospital (Kyoto, Japan) and Fujita Health University Hospital (Aichi, Japan), with written informed consent from the patients and approval from the ethics committee."

  Corrected "Patient data were collected and provided in an anonymized form by the Rakuwakai Otowa Hospital (Kyoto, Japan) and Fujita Health University Hospital (Aichi, Japan), with written informed consent from the patients. Ethical approval for this study was granted by the Research Ethics Committee of The University of Tokyo, the Ethics Committees for Human Research of Rakuwakai Otowa Hospital, and the Ethics Review Committee of Fujita Health University."
- Page 19, lines 476–481: The original text could be confusing and therefore has been corrected.

Original "However, an exception was made for Patient 2, whose left and right anterior cerebral arteries were extremely close, as depicted in Fig 3. As it was impossible to recognize the ACoA on CT images, we assumed uncertainty of 0.1–2.6 mm in its diameters, which includes the possibility that the artery is absent."

Corrected "However, an exception was made for Patient 2, as the ACoA was not recognized on CT images of this patient, suggesting hypoplasia of the ACoA. Nevertheless, we could not rule out the possibility that the ACoA, hidden between the extremely close presence of the left and right anterior cerebral arteries, might have failed to resolve on the images (Fig 3). Therefore, we assumed uncertainty of 0.1–2.6 mm in the ACoA diameter, thereby including the possibility of its absence as well as presence."

- Some minor edits have been made throughout the manuscript to improve clarity. All changes have been tracked in the highlighted version of the revised manuscript.
- To conform to the journal policy related to the file size limit, S1 File (originally attached as ZIP file) has been made available on Zenodo, and a link to the file has been provided in the manuscript. Data and Code Availability now states:

  "All relevant data are available on Zenodo (https://doi.org/10.5281/zenodo.6557448), except for medical images that could potentially identify or reveal sensitive patient information."
- Per the journal policy on revised manuscripts, figures have been removed from the manuscript file (except for captions) and attached as separate files.
